# Gene discovery for the bark beetle-vectored fungal tree pathogen *Grosmannia clavigera*

**DOI:** 10.1186/1471-2164-11-536

**Published:** 2010-10-04

**Authors:** Uljana Hesse-Orce, Scott DiGuistini, Christopher I Keeling, Ye Wang, Maria Li, Hannah Henderson, T Roderick Docking, Nancy Y Liao, Gordon Robertson, Robert A Holt, Steven JM Jones, Jörg Bohlmann, Colette Breuil

**Affiliations:** 1 Department of Wood Science, University of British Columbia, Vancouver, Canada; 2 Michael Smith Laboratories, University of British Columbia, Vancouver, Canada; 3 BC Cancer Agency Genome Sciences Centre, Vancouver, Canada

## Abstract

**Background:**

*Grosmannia clavigera *is a bark beetle-vectored fungal pathogen of pines that causes wood discoloration and may kill trees by disrupting nutrient and water transport. Trees respond to attacks from beetles and associated fungi by releasing terpenoid and phenolic defense compounds. It is unclear which genes are important for *G. clavigera*'s ability to overcome antifungal pine terpenoids and phenolics.

**Results:**

We constructed seven cDNA libraries from eight *G. clavigera *isolates grown under various culture conditions, and Sanger sequenced the 5' and 3' ends of 25,000 cDNA clones, resulting in 44,288 high quality ESTs. The assembled dataset of unique transcripts (unigenes) consists of 6,265 contigs and 2,459 singletons that mapped to 6,467 locations on the *G. clavigera *reference genome, representing ~70% of the predicted *G. clavigera *genes. Although only 54% of the unigenes matched characterized proteins at the NCBI database, this dataset extensively covers major metabolic pathways, cellular processes, and genes necessary for response to environmental stimuli and genetic information processing. Furthermore, we identified genes expressed in spores prior to germination, and genes involved in response to treatment with lodgepole pine phloem extract (LPPE).

**Conclusions:**

We provide a comprehensively annotated EST dataset for *G. clavigera *that represents a rich resource for gene characterization in this and other ophiostomatoid fungi. Genes expressed in response to LPPE treatment are indicative of fungal oxidative stress response. We identified two clusters of potentially functionally related genes responsive to LPPE treatment. Furthermore, we report a simple method for identifying contig misassemblies in *de novo *assembled EST collections caused by gene overlap on the genome.

## Background

The ophiostomatoid fungus *Grosmannia clavigera *(Robinson-Jeffrey and Davidson) is a fungal pathogen that discolours wood, and kills pine host trees by disrupting the flow of nutrients and water in phloem and sapwood [1,2]. In its ecosystem, *G. clavigera *is vectored between hosts by the mountain pine beetle (MPB, *Dendroctonus ponderosae*). This pathogen can kill lodgepole pine (*Pinus contorta*) when manually inoculated under the bark at a high enough concentration [3,4]. Like many bark beetle associated fungi, *G. clavigera *produces slimy spores that stick to the exoskeleton of the insects, but the fungus is also present in the beetle mycangia and alimentary canal [5]. During tree colonization, the beetles spread fungal spores throughout their galleries below the bark. The spores germinate and fungal hyphae colonize the phloem and sapwood of the tree. Fungi benefit the beetles by improving the host environment for the beetle progeny, and serving as food for the larvae and the teneral adult beetles [6]. In addition, *G. clavigera *may counteract tree defenses that are activated during bark beetle attacks.

Conifer trees respond to beetle attacks or fungal inoculation by releasing resin from pre-formed and inducible traumatic resin ducts, inducing the synthesis of phenolic compounds in phloem parenchyma cells, and forming a wound periderm tissue [7]. The main constituents of conifer resin are terpenoids, many of which have insecticidal and fungicidal properties [8,9]. Ophiostomatoid fungi can decrease the concentration of terpenoids when inoculated on sapwood [10-12]. However, the molecular and biochemical mechanisms involved in these processes are unknown. Previously, we analyzed a small set of expressed sequence tags (ESTs) from *G. clavigera *and found putative gene candidates that may be involved in terpenoid detoxification [13]. Lodgepole pine also constitutively produces a variety of phenolic compounds, including flavonoids and tannins [14-17]. Many of these chemicals inhibit the growth of fungal pathogens [18]. To detoxify phenolic plant defense compounds, fungi produce a variety of enzymes such as phenol oxidases that polymerize phenolics [19], peroxidases that degrade polymeric phenolic structures [20], and glucosidases and glucuronidases that are involved in metabolism of phenolic glycosides [21,22]. As well, fungi may release extracellular proteins that bind to toxic phenolics preventing their interaction with the fungal cell wall [23]. Whether *G. clavigera *encodes and expresses genes necessary for detoxification of phenolic compounds has not been determined.

The recently published genome of *G. clavigera *is the first reference genome for an insect-vectored fungal tree pathogen [24]. The genome sequence provides a fundamental resource for identifying fungal genes important for symbiotic interactions with insect and tree hosts. ESTs support gene discovery and gene structure annotation. To cover a broad spectrum of expressed genes, we extended the previous collection of 5,950 ESTs [13], and sequenced the 5' and 3' ends of 25,000 cDNA clones from normalized and non-normalized cDNA libraries of eight *G. clavigera *isolates grown in various conditions. We conducted a *de novo *EST assembly and used the resulting set of unique transcripts (unigenes) to verify the assembly of the *G. clavigera *genome [24]. As well, we used the genome sequence to address two problems associated with *de novo *CAP3 EST assembly: 1) incomplete assembly of ESTs from multiple transcripts of a given gene into a single contig due to incomplete splicing, splice variants, incomplete read overlap, and sequencing errors; and 2) incorrect assembly of ESTs from transcripts of different genes as a result of sequence similarity.

Here we describe the *G. clavigera *unigene dataset, and discuss fungal genes upregulated in two biological states that are important for *G. calvigera*-MPB-pine interactions: the vectored non-germinating asexual spores, and mycelia treated with lodgepole pine phloem extract (LPPE), which contains tree defense chemicals. Furthermore, we show that overlap of neighboring genes on the genome was the major source for contig misassemblies in this *de novo *EST assembly, and describe a simple method for identifying such cases even in absence of a sequenced genome.

## Results

### 1 EST analysis

To identify a broad spectrum of *G. clavigera *genes necessary for fungal growth we grew eight isolates (Table 1) on five media, and for one medium we treated the mycelia with LPPE (Table 2). We also harvested spores from the reference isolate SLKW1407 that we had used to sequence the *G. clavigera *genome [24]. To maximize sequencing efficiency we pooled the media and LPPE treatments, generating three isolate/treatment combinations with the reference isolate, and one isolate/treatment combination that contained the seven closely related *G. clavigera *isolates (Table 3). Furthermore, we prepared normalized cDNA samples for three isolate/treatment combinations. We then constructed seven unidirectional, full-length enriched cDNA libraries and sequenced a total of 25,000 cDNA clones from both 5' and 3' ends (Table 3). After trimming vector and low quality sequences the average PHRED 20 read length was 693 bp. This resulted in 44,288 high quality ESTs (NCBI dbEST GT571598-GT615878). After adding 5,950 quality-filtered ESTs from previous analyses with the reference isolate [13], we reverse-complemented 3' reads, removed polyA tails, and discarded sequences with long mononucleotide stretches. The resulting dataset contained 50,167 high quality ESTs.

**Table 1:** *G. clavigera *strains isolated from *Pinus *species used for cDNA library construction

ID	Isolate	ATCC/UAMH accession	Isolation origin	Host	Isolated from	Year
1	SLKW1407	UAMH11150	BC/Kamloops	*P. contorta*	Gallery	2003
2	ATCC18086	ATCC18086	BC/Cache Creek	*P. ponderosa*	Sapwood	1965
3	200-1-14	UAMH11151	BC/Kamloops	*P. contorta*	Sexual spore	2004
4	DPLKGT1B	UAMH11152	BC/Kelowna	*P. contorta*	MPB body	2007
5	H55	UAMH11153	BC/Houston	*P. contorta*	MPB body	2003
6	B5	UAMH11154	Alberta/Banff	*P. contorta*	MPB body	2003
7	B10	UAMH11155	Alberta/Banff	*P. contorta*	MPB body	2003
8	DPCHMC3	*	Alberta/Cypress Hills	*P. contorta*	MPB mycangia	2007

*Breuil culture collection, Dept. of Wood Science, University of British Columbia, Vancouver, Canada

**Table 2:** Media and treatment for growing *G. clavigera *mycelium for cDNA library construction

Media	composition
wood	10 g/plate lodgepole pine sawdust
starch	0.17% YNB, 0.1% PHP, 0.3% asparagine, 1% starch
organic nitrogen	0.17% YNB, 0.1% PHP, 0.3% asparagine, 1% maltose
inorganic nitrogen	0.17% YNB, 0.1% PHP, 0.3% NaNO3, 1% maltose
olive oil	0.17% YNB, 0.1% PHP, 0.3% asparagine, 1% (v/v) olive oil emulsified in 0.5% tergitol (olive oil and tergitol were mixed and autoclaved separately before being added to the media)

**LPPE treatment**	Cultures were grown on organic nitrogen medium for 48 h and then sprayed with LPPE

All media were solidified with 1.5% granulated agar. LPPE = lodgepole pine methanol extract, YNB = yeast nitrogen base, PHP = potassium hydrogen phthalate

**Table 3:** cDNA libraries from four *G. clavigera *isolate-treatment combinations and numbers of high quality ESTs derived from each library.

Library	Iso-lates	Media/treatment combinations	Norma-lized	Primary titre (cfu/ml)	ESTs sequenced	ESTs HQ (%)
OCL01	1	W:S:ON:IN:OO	No	2.1 × 10^6^	6,144	5,272 (86)
OCL02	1	W:S:ON:IN:OO	Yes	2.1 × 10^5^	15,360	14,075 (92)

OCL03	1	ON+LPPE	No	4.0 × 10^5^	6,144	5,618 (91)
OCL04	1	ON+LPPE	Yes	2.5 × 10^5^	9,216	7,794 (85)

OCL05	2-8	W:S:ON:IN:OO:ON+LPPE	No	2.0 × 10^6^	3,072	2,916 (95)
OCL06	2-8	W:S:ON:IN:OO:ON+LPPE	Yes	9.0 × 10^4^	3,072	2,819 (92)

OCL08	1	Sp	No	1.3 × 10^6^	6,912	5,794 (84)

For the libraries OCL01-OCL06, mycelial cultures from each of the eight isolates were grown on five different media, and for one medium the mycelia were treated with LPPE (W = wood, S = starch, ON = organic nitrogen, IN = inorganic nitrogen, OO = olive oil, LPPE = treatment with lodgepole pine methanol extract). After extracting total RNA separately from each of the 48 isolate/media/LPPE-treatment variants and from the spore sample (Sp), equal amounts of total RNA were pooled to obtain four isolate/treatment combinations. From these pooled samples we purified poly(A+) mRNA and generated cDNA. All three non-spore cDNA samples were divided into two fractions, one of which was normalized. The resulting seven cDNA samples were used for library construction. HQ = high quality.

### Unigene assembly and unigene-locations on the genome

We assembled the 50,167 ESTs into unigenes and determined their locations on the *G. clavigera *genome. Assembly resulted in 8,724 unigenes (2,459 singletons and 6,265 contigs) that joined 91% of the 21,391 5'-3' read pairs. Most of the unigenes (97%) mapped to the genome at high stringency (>80% sequence similarity; >80% unigene alignment length), while 219 unigenes mapped with a quality below this threshold, and 48 unigenes did not align to the genome. We evaluated the contig assembly using two methods. First, we aligned the unassembled reads to the genome and tested whether all reads from a contig would map to the same genome location as the contig. For 74 contigs there was disagreement. Manual inspection showed that 28 were correctly assembled and could be matched to an NCBI protein sequence, leaving 46 (0.5%) potentially misassembled unigenes. Second, we searched for contigs containing forward and reverse reads (FR contigs) despite unidirectional orientation of the assembled reads. We identified 821 such contigs, and in most cases found indications for transcript overlap between neighboring genes encoded on opposite genome strands. For all but 21 (2.6% of 821) FR contigs, the unassembled reads mapped to the same genome location as their respective contig aligning, however, to both strands of the genome. We manually inspected 50 FR contigs and found that for 35 of them the reverse assembled reads belonged to an adjacent gene model encoded on the opposite strand. For the remaining 15 FR contigs all reads mapped to the same region on the genome, but we found no evidence for a neighboring gene.

We generated 6,467 unigene-locations (ULs) on the *G. clavigera *reference genome based on strand-specific unigene overlap and linking information from unassembled 5'-3' read pairs (Additional file 1: Table S1). Of these, 812 ULs (13%) overlapped with another UL encoded on the opposite strand of the genome, with a 260 bp median overlap length. However, FR contig analysis suggested that UL overlap occurs more frequently. For the 814 ULs with FR contigs, UL analysis identified 170 overlapping ULs (21%). Manual inspection of genome locations with FR contigs indicated this fraction to be ~70% (i.e. ~570 ULs). This suggested that overall ~1212 *G. clavigera *ULs overlapped.

### Genes represented in the unigene dataset

We predicted ORFs for all but five of the 8,724 unigenes; 1,584 of these unigenes (18%) were full length, 4,806 were truncated at the 5' end, and 771 were truncated at the 3' end. Seventy-three percent of the unigenes were similar to NCBI protein sequences (min e-value 1.0 × 10^-4^). For these unigenes ORF predictions were based on the best BLAST match, and we found that for 1,115 of them the best predicted ORF was not the longest ORF. We also noted that only 8% of the correctly assembled unigenes had a significant protein match on the reverse strand, while 56% of the FR contigs had a significant hit on the minus frame. Using InterProScan and KAAS, we assigned InterPro IDs, Gene Ontology terms, and K-numbers with corresponding BRITE classifications to 3,730 (43%), 2,504 (29%) and 1,530 (18%) unigenes, respectively. The K-number annotated unigenes belonged to 1,439 ULs.

**Figure 1:**
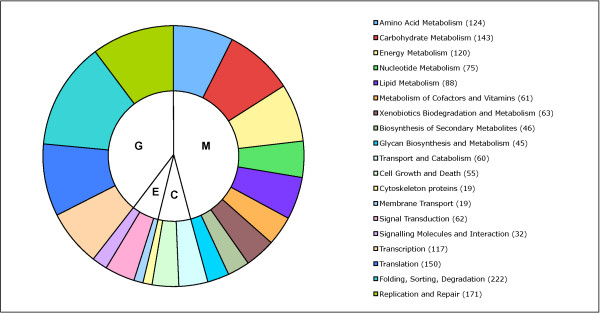
**Numbers of unigene-locations involved in metabolic pathways (M), cellular processes (C), environmental information processing (E), and genetic information processing (G) based on KEGG-BRITE annotations**.

Hierarchical classification of the unigenes using KEGG-BRITE allowed mapping ULs to pathways and infer higher-order functions (Figure 1, Additional file 1: Table S1). Nearly half of the 1,439 ULs that were annotated with K-numbers encoded proteins from metabolic pathways, including amino acid metabolism, carbohydrate metabolism, energy metabolism, as well as lipid and nucleotide metabolism. Proteins encoded by 575 ULs are potentially involved in genetic information processing (i.e. transcription, translation, replication and DNA repair). Furthermore, we verified that this *G. clavigera *unigene collection covers essential metabolic pathways. Using reciprocal BLAST analysis we identified all genes of the ergosterol pathway, all but one of the genes of the citrate and pentose phosphate cycles, and 59 of 95 genes necessary for primary amino acid biosynthesis.

### 2 Differential gene expression

**Figure 2:**
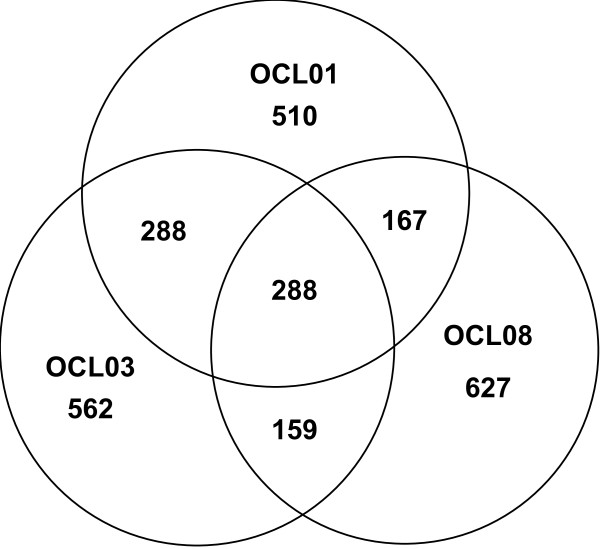
**Numbers of unique and shared transcripts in the non-normalized mycelial culture (OCL01), LPPE (OCL03), and spore (OCL08) cDNA libraries of *G. clavigera***.

**Figure 3:**
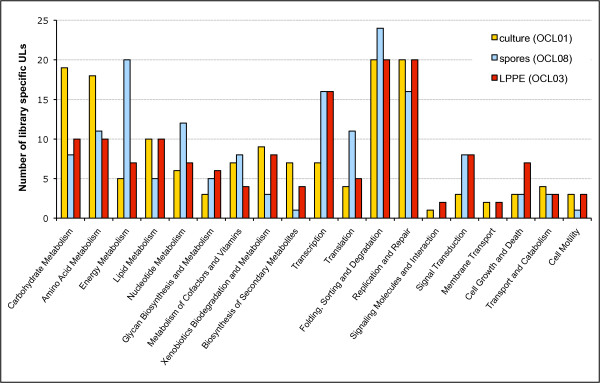
**Library specific unigene-locations (ULs) from the non-normalized mycelial culture (OCL01), LPPE (OCL03), and spore (OCL08) cDNA libraries classified based on KEGG-BRITE annotations (n_OCL01 _= 141, n_OCL03 _= 126, n_OCL08 _= 156)**.

We characterized gene expression in the *G. clavigera *reference isolate (SLKW1407) by assessing EST frequencies in the non-normalized cDNA libraries from mycelial culture (OCL01), spores (OCL08), and cultures exposed to LPPE treatment (OCL03). Transcripts for the majority of ULs (1699; 65%) appeared to be library specific, 614 ULs (24%) contained transcripts from two libraries, and only 288 ULs (11%) contained reads from all three libraries (Figure 2). To identify genes associated with processes that were overrepresented under any of the three conditions tested, we analyzed the set of library-specific ULs that were annotated with K-numbers and assigned to KEGG Pathways (Figure 3). Table 4 shows selected pathways for which numbers of specifically expressed genes differed between the three cDNA libraries. Among the ULs expressed only in the mycelial culture library we found twice as many carbohydrate and amino acid metabolism genes than in the other libraries. Spore-library specific ULs encoded a higher variety of proteins necessary for oxidative phosphorylation, nucleotide metabolism, and translation. In the LPPE library, genes for signal transduction and N-glycan biosynthesis were specifically expressed. Below we describe ULs that were identified as differentially expressed by reciprocal comparison of read frequencies of the three non-normalized cDNA libraries.

**Table 4:** Numbers of genes in selected KEGG pathways matched by ULs specific to cDNA libraries OCL01 (mycelial culture), OCL03 (LPPE), and OCL08 (spores)

Pathway	OCL01	OCL03	OCL08
Oxidative phosphorylation	1	3	14
Amino sugar and nucleotide sugar metabolism	6	2	1
Starch and sucrose metabolism	5	1	0
Phenylalanine, tyrosine and tryptophan biosynthesis	5	1	1
N-Glycan biosynthesis	0	3	2
High-mannose type N-glycan biosynthesis	2	0	1
O-Mannosyl glycan biosynthesis	0	1	1
Biosynthesis of terpenoids and steroids	3	6	0
Pyrimidine metabolism	3	6	8
Purine metabolism	4	4	11
RNA polymerase	0	2	7
Spliceosome	3	9	5
Aminoacyl-tRNA biosynthesis	2	1	6
MAPK signaling pathway - yeast	0	4	1
Cell cycle - yeast	1	6	2

### Spores (OCL08) *vs *mycelial culture (OCL01)

Of the 2,039 ULs with reads from the libraries OCL01 and/or OCL08, 66 ULs had significantly (p < 0.05) higher read frequencies in the spore than in the mycelial library (Additional file 1: Table S1). Of these, 11 ULs (355, 444, 2425, 2447, 2745, 3423, 3838, 4185, 4783, 5218, 5775) encoded heat shock proteins (HSPs) and other protein chaperones/folding catalysts (e.g. cyclophillin D). Other ULs that were overexpressed in spores matched proteins involved in energy metabolism and ATP-dependent cellular processes: Ca^2+ ^transporting ATPase (UL 904), F-type H+-transporting ATPase subunit epsilon (UL 2399), arsenite-transporting ATPase (UL 4670), ATP-dependent Clp protease (UL 5775). For 73 ULs read frequencies were significantly higher in the mycelial than in the spore libraries (Additional file 1: Table S1). A high fraction of ULs that were overexpressed in mycelial culture encoded proteins important for carbohydrate and amino acid metabolism: four glycolytic enzymes (ULs 2601, 4586, 6279, 6391), a glucose transporter (UL 5108), a subtilase (UL 4937), and proteins involved in methionine (ULs 1740, 6245) and melanin (UL 4533) biosynthesis.

### LPPE treatment (OCL03) *vs *mycelial culture (OCL01)

**Figure 4:**
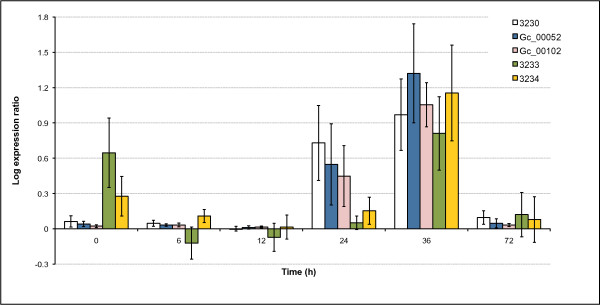
**qRT-PCR results showing the expression levels of five unigene-locations at six time points after LPPE treatment relative to their expression levels in cultures of *G. clavigera *treated with a methanol control solution**. (3230 - MFS-transporter, Gc_00052 - P450, Gc_00102 - P450 reductase, 3233 - steroid monooxygenase, 3234 - beta-lactamase)

We identified eight ULs (85, 588, 1906, 2850, 3233, 3525, 4003, 4803) that were significantly (p < 0.05) overexpressed in the LPPE treated culture library (Additional file 1: Table S1). We analyzed the genomic neighborhoods of these eight ULs and found that two ULs (annotated as a steroid monoxygenase and a cupin domain protein) co-localized with other ULs that were expressed only in the LPPE treated libraries, and so may be functionally related. The first cluster (Table 5) contained the steroid monoxygenase and ULs encoding a beta-lactamase (whose ESTs originated from the normalized LPPE library), an MSF transporter, a cytochrome P450, and a P450 reductase. RT-PCR confirmed that transcript abundance for all five of these clustered ULs were increased in response to LPPE treatment at the 36 h time point (Figure 4). Reciprocal BLAST searches indicated that this cluster is not conserved in *Aspergillus *spp., *Fusarium *spp., *Magnaporthe grisea*, and *Neurospora crassa*. However, in *Aspergillus nidulans*, the putative orthologues of the cytochrome P450 (AN5837) and the P450 reductase (AN5838) are located next to each other. The second cluster (Table 6) contained the cupin domain protein and ULs for an S15 family peptidase, an unknown protein, an aromatic ring-opening dioxygenase, a PutA family dehydrogenase, and a short chain dehydrogenase. Three ULs of this cluster were specific to the LPPE treatment libraries. This cluster was not conserved in the above fungal species. For 11 ULs (602, 769, 2601, 3744, 3874, 4487, 4533, 5218, 5833, 6220, 6391), read frequencies were significantly (p < 0.05) lower in the LPPE library than in the mycelial library (Additional file 1: Table S1). Eight of these, including the ULs for trehalase, scytalone dehydratase 1, and HSP90 were also downregulated in the spore library. Analysis of the genomic regions flanking these ULs did not indicate clustering of functionally related genes.

**Table 5:** Unigene-locations of cluster 1, which is potentially involved in response to lodgepole pine phloem extract

Unigene-location	Best annotated protein match from the NCBI nonredundant protein database	e-value	p-value*
3230	MFS sugar transporter [*Aspergillus flavus*]	4E-92	ns
3231	related to ARCA protein [*Neurospora crassa*]	4E-38	ns
Gc_00052	benzoate 4 monooxygenase cytochrome P450 [*Neosatoria fisherii*]	1E-165	ns
Gc_00102	NADPH-cytochrome P450 reductase (CprA) [*Aspergillus fumigatus*]	4E-143	ns
3232	small s protein [*Podospora anserina*]	7E-42	ns
3233	steroid monooxygenase [*Aspergillus flavus*]	1E-104	0.02
3234	beta-lactamase family protein [*Pyrenophora tritici-repentis*]	2E-58	Ns

* Fisher's exact p-value for the comparative analysis on transcript abundance between the mycelial culture (OCL01) and LPPE (OCL03) libraries.

**Table 6:** Unigene-locations of cluster 2, which is potentially involved in response to lodgepole pine phloem extract

Unigene-location	Best annotated protein match from the NCBI nonredundant protein database	e-value	p-value*
3999	X-Pro dipeptidyl-peptidase (S15 family) protein [*Neosartorya fischeri*]	8E-113	ns
4000	predicted protein [*Sclerotinia sclerotiorum*]	1E-52	ns
4001	aromatic ring-opening dioxygenase family protein [*Talaromyces stipitatus*]	7E-81	ns
4002	PutA family dehydrogenase [*Talaromyces stipitatus*]	2E-13	ns
4003	cupin domain protein [*Neosartorya fischeri*]	3E-50	0.03
4004	short chain dehydrogenase [*Aspergillus fumigatus*]	4E-44	ns

* Fisher's exact p-value for the comparative analysis on transcript abundance between the mycelial culture (OCL01) and LPPE (OCL03) libraries.

## Discussion and Conclusions

ESTs can be assembled *de novo*, or, when a high-quality reference genome sequence is available, by mapping sequence reads to the genome and assembling them by genome location. We conducted a *de novo *EST assembly to assess the quality of the *G. clavigera *genome sequence [24]. For this, we applied CAP3, a widely used EST assembly program that is a component of recent assembly pipelines [25,26]. Of the 8,457 unigenes, 97% mapped to the genome at high stringency (>80% sequence similarity; >80% unigene alignment length). Only 0.5% of the unigenes contained reads from distant genome locations, indicating either unigene or genome misassemblies. These results show that both the assembled genome sequence and the unigene assembly were of high quality. The unigene collection derived from 50,167 high quality ESTs allowed identification of 6,467 ULs. In a separate analysis of the *G. clavigera *genome sequence we estimated ~9,000 genes (unpublished results). Based on this number the unigene set represents ~70% of the *G. clavigera *genes. Unigene annotation showed that our dataset covers in depth major metabolic pathways for general and essential processes, and provides sequence information on genes that may be unique to *G. clavigera*. These data will be a useful resource for future research on this pathogen, in particular for its symbiotic association with mountain pine beetle and interaction with the chemical defenses of host trees.

Antisense transcripts may be involved in controlling gene expression [27,28]. We estimated that ~19% of *G. clavigera *ULs overlap with another UL from the opposite genome strand, and so potentially represent antisense transcripts. Our analysis indicated, that in most cases contigs containing forward and reverse assembled reads despite unidirectional read orientation (FR contigs) were assemblies of transcripts from overlapping genes. Therefore, FR-filtering can help identify assembly problems as well as potential antisense transcripts in de novo assembled unigene collections.

Control of spore germination may be an important factor in *G. clavigera *bark beetle symbiosis and tree pathogenicity. Transcript analysis of the spore library indicated that early germination processes may have been induced in our spore samples. We identified several protein chaperones, including two HSP70s (ULs 355, 2425) and one cyclophillin D (UL 3423) that showed significant similarity to the *A. nidulans *proteins XP_663614, XP_662733, and XP_662187, respectively. Proteome analyses revealed that these proteins accumulated in *A. nidulans *within 30 to 60 minutes after conidia germination [29]. Other ULs with high transcript frequencies in spores of *G. clavigera *encoded proteins involved in energy metabolism and protein biosynthesis, which is consistent with respiration and protein biosynthesis activated early in germination [30]. The genes identified in this analysis may serve as markers for early spore germination. However, analysis of gene expression in resting and germinating *G. clavigera *spores requires further work.

Genes whose transcripts were over-represented in response to LPPE treatment suggested a fungal oxidative stress response caused by host phenolic or other defense compounds present in the phloem extract [31,32]. For example, we observed a large number of ESTs for Cu/Zn superoxide dismutase (SOD) in the LPPE treatment library (UL 1906). SOD catalyses the conversion of superoxide radicals to molecular oxygen. SOD1 mutants of *N. crassa *are sensitive to superoxide-generating compounds and have a high rate of spontaneous mutations [33]. In contrast, deleting SOD1 did not change the sensitivity of *Claviceps purpurea *to paraquat and did not affect its pathogenicity [34], indicating that other detoxifying systems may be involved. Interestingly, our EST data revealed a second Cu/Zn superoxide dismutase (UL 3288), which does not appear to be upregulated by LPPE treatment, suggesting that the two SODs may have different functions. Similarly, a peroxidase (UL 588) overexpressed in the LPPE treatment library may participate in scavenging reactive oxygen species. Alternatively, it may be involved in detoxification of phenolic compounds, as previous studies have shown that peroxidases can oxidize phenolic substrates and cleave aromatic ring structures [20,35]. EST frequencies in the LPPE treatment library were also high for a nitroreductase (UL 4803), but functions of this gene in host colonization are uncertain. Bacterial nitroreductases can detoxify nitrosubstituted compounds [36], including nitrophenols [37] in reactions that produce superoxides and induce oxidative stress [38,39]. In *S. cerevisiae *two nitroreductases have been identified [40], one of which appears to act in the lipid-signaling pathway. Mutants for these enzymes showed extreme sensitivity to nitrosative substances and have reduced superoxide dismutase activity [41]. The authors hypothesized that the nitroreductases may modulate antioxidant enzymatic activities in yeast.

In filamentous fungi, functionally related genes and genes involved in niche adaptation can occur in clusters. For example, secondary metabolite genes in *A. fumigatus *[42] and genes encoding secreted proteins in *Ustilago maydis *[43] are clustered. For *G. clavigera*, we identified two clusters of putatively functionally related genes that were upregulated after LPPE treatment. The first cluster contained five genes: a steroid monooxygenase, a P450, a P450 reductase, a beta lactamase, and an MFS transporter. The steroid monooxygenase and the P450 may participate in detoxification of LPPE compounds by adding or altering functions of metabolites, similar for example to the P450s involved in fungal detoxification of pisatin [44]. The conserved co-localization of the P450 and the P450 reductase has been noted in *Aspergillus *species [45] and led to the hypothesis that this reductase may be specific for the co-localized P450. The observed co-regulation of these two *G. clavigera *genes in response to LPPE treatment is consistent with this hypothesis. Whether the gene showing high similarity to beta lactamase is involved in ring cleavage of any LPPE metabolite remains to be tested in future work. The second cluster contained an extradiol ring-cleavage dioxygenase and two oxidoreductases, and may be involved in degrading aromatic compounds such as phenolics. Further analyses on genes in these two clusters are necessary to confirm functions and relationships.

## Methods

### Fungal isolates and culture conditions

The eight fungal isolates used in this study are listed in Table 1. To induce a broad spectrum of genes, cultures from each isolate were grown on five different media (Table 2) that varied in nitrogen and carbon sources. For this, we first obtained young mycelial cultures by inoculating plates containing 1% malt extract agar (MEA, Oxoid, England) overlaid with cellophane with a spore suspension (12 plates/isolate, 5 × 10^5 ^spores/plate), and incubating them at ambient conditions for 48 h. Then, we transferred the cellophane with the young mycelia to new plates that contained the different media (2 plates/isolate/medium). The cultures were grown for four days, and then harvested for RNA extraction, pooling the mycelia from the two plates of each isolate/medium combination.

To identify genes expressed in mycelial culture in response to lodgepole pine phloem extract (LPPE) we grew fungal cultures from the eight isolates on organic nitrogen media (as described above) for 48 h, sprayed them with 200 μl of crude LPPE, and harvested the mycelia for RNA extraction 48 h after initial exposure to LPPE. LPPE was prepared as follows: a lodgepole pine bolt from a freshly cut tree was frozen at -20°C and cut into disks. The frozen phloem was separated and ground in a mill with liquid nitrogen. The powder was extracted in 80:20 methanol:water (2.5 ml/g) and sonicated at 4°C for 2 h. After centrifugation, the supernatant was removed and concentrated by 1/3 under a gentle flow of nitrogen gas (final concentration ~50:50, MeOH:H_2_O). This concentrated crude extract was stored at -20°C. To ensure uniform treatment of all cultures, a single preparation of LPPE was used for the experiment.

For the spore library, cultures of the reference isolate SLKW1407 were grown for seven days on 1% MEA. Spores were harvested in 5 ml water and filtered through a BD Falcon Cell strainer (40 μm) to remove mycelial debris from the spore collection. The spore suspension was centrifuged at 5,000 rpm for 20 min, the pellets were transferred to 1.5 ml microtubes and centrifuged again for 2 min at 14,000 rpm. These tubes were stored at -80°C until RNA extraction.

### RNA isolation, cDNA library construction, cDNA sequencing

Total RNA was isolated separately from each of the 48 isolate/media/LPPE treatment variants and the spores, respectively, using the RNeasy Mini Plant RNA isolation kit (Qiagen, Mississauga, ON, Canada). The total RNA samples were then treated with DNaseI (Fisher Scientific, Ottawa, ON, Canada), analyzed for quality by spectrophotometer and agarose gel analysis, and quantified. To generate comparable treatments for the reference isolate, and to ensure high sequencing efficiency, we pooled equal amounts of total RNA from the different isolate/media/LPPE treatment variants and prepared four isolate/treatment combinations: three treatments with the reference isolate, and one pool containing total RNA from the other seven isolates (Table 3). From the resulting four RNA samples we purified poly(A+) mRNA using the Oligotex mRNA purification kit (Qiagen), and generated first strand cDNA using SuperScript III reverse transcriptase (Invitrogen, Carlsbad, CA, USA), CDS-3M primer (Evrogen, Moscow, Russia), and SMART IV Oligonucleotide (Clontech, Mountain View, CA, USA). Second strand cDNA was prepared by long distance-PCR with Phusion Hot Start DNA Polymerase (Finnzymes, Espoo, Finland). The cDNA samples were split into two fractions and one fraction was normalized using the TRIMMER-DIRECT cDNA normalization kit (Evrogen). For library construction, normalized and non-normalized cDNA was digested with SfiI and size fractionated. The fractions >500 bp were directionally cloned into the SfiI-digested pDNR-LIB vector (Clontech). A set of 25,000 clones randomly selected from all libraries were partially sequenced on a 3730XL DNA analyzer (Applied Biosystems, Carlsbad, CA, USA) from the 5' and 3' ends using the -21 M13 forward and M13 reverse primers, respectively. Sequencing was done at the British Columbia Cancer Agency Genome Sciences Centre (Vancouver, BC, Canada).

### qRT-PCR analysis

For quantitative real time PCR (qRT-PCR) we grew the reference isolate SLKW1407 on organic nitrogen media (as described above) and treated cultures with either LPPE, or a methanol control solution (50:50, MeOH:H_2_O). We harvested mycelia at 0, 6, 12, 24, 36, 48, and 72 h after the beginning of treatment, and proceeded with qRT-PCR analysis following the protocol described by DiGuistini et al. [13]. For each time point we prepared three biological and three technical replicates. The primers used to amplify the transcript of interest are shown in Table 7. Data collection and statistical analysis were conducted with the Roche CFX 96-real-time PCR detection system (Roche, Quebec, CA).

**Table 7:** Primers used for quantitative RT-PCR analysis of unigene locations (ULs)

Target UL	Primer name	5'-3' sequence
3230	R1718-F1	GTG TCC TCC ACC TTC CTC ACC
	R1718-R1	CGT GAC TCC CTT GAC TTC TGG G
Gc_00052	Gc_0052-F1	GCT CTC TCT TTT GCC GGC GGA
	Gc_0052-R1	GAG CCG GCC AGC GTT GAG TAA
Gc_00102	Gc_00102-F1	TCG GAC GGA CTG CAA ACG CG
	Gc_00102-R1	CGA GCC CCA GAA AAG GAC GAC
3233	R1719-F1	CTC AGC AAC GGT CCA ACC TC
	R1719-R1	GTG CTT CTT CCA CTT GCG GG
3234	F1718-F3	GAGCTGCTGACGCTCGATAA
		F1718-R3	ACCTGACTGCTGTCGTCCAT

### EST assembly

We processed chromatograms and trimmed low quality sequences using PHRED [46], requiring a minimum of 100 bp with quality scores above PHRED 20. Using cross-match (http://www.phrap.org/), we removed vector sequences and filtered the remaining sequences for *Escherichia coli*. To account for possible contamination from the lab environment we also screened for *Saccharomyces cerevisiae*, *Agrobacterium*, and *Aspergillus *spp. To the resulting set of 44,288 quality-filtered reads we added 5,950 quality-filtered *G. clavigera *reads (isolate SLKW1407) from previous work [13]. Since the cDNA libraries contained directionally cloned inserts, sequences obtained using the M13 reverse primer (3' reads) had a 3'-5' orientation with respect to the original mRNA. These 3' reads were reverse-complemented to produce a 5'-3' oriented read collection. Then we removed the polyA tails, which interfere in contig assembly, and discarded sequences with long mononucleotide stretches. The resulting set of 50,167 high-quality reads was assembled with CAP3 [47] using default settings except for a minimum overlap of 40 bp and a minimum identity of 95%.

Because the reads were 5'-3' oriented prior to assembly, most of the unigenes had a 5'-3' orientation. This was verified by comparing the input fasta files and the assembler's ACE-format output files. Then, we screened the ACE file for contigs that contained reads in both orientations. Because proteins are encoded on the forward strand of the transcript, such contigs potentially represent misassembled unigenes.

### Unigene-locations on reference genome

The unigenes were mapped against the *G. clavigera *genome [24] using BLAT [48]. For each unigene, we selected the best hit to the genome, and recorded sequence similarity (%) and unigene alignment length (%). Since all unigenes were 5'-3' oriented they mapped to the genome strand that encoded the respective gene. To group unigenes that represented the same gene, we generated unigene-locations (ULs) based on strand-specific overlap of unigenes on the genome, and linking information from unassembled 5'-3' clone pairs. We verified unigene assembly by mapping all reads to the genome with BLAT and testing whether the unassembled reads of a contig mapped to the same genome location as the contig.

### Unigene annotation

For annotation, all unigenes were compared to the non-redundant NCBI protein database using BLAST (NCBI, Bethesda, MD, USA; min e-value 1.0 × 10^-4^). We used custom Perl scripts to capture the best BLAST hit on the forward frame and determine the most likely open reading frame (ORF) for each sequence. For the latter, all ORFs longer than 25 amino acids were identified using CLC Genomics Workbench (CLC bio, Aarhus, Denmark). If the sequence had a significant BLAST match on the forward frame, the best ORF covering that match and the most likely transcription start and stop were determined, keeping track of 5' and 3' ORF truncations. If no BLAST match was available, the longest ORF on the forward frame was selected for the sequence. In addition, unigenes were annotated with InterPro IDs and Gene Ontology terms using InterProScan (EBI, Cambridge, UK), and assigned K-numbers with corresponding KEGG-BRITE classifications using KAAS [49]. To evaluate unigene representation in essential metabolic pathways, we conducted a reciprocal BLAST analysis on the translated *G. clavigera *unigene ORFs against the annotated *Magnaporthe grisea *and *Aspergillus fumigatus *protein datasets downloaded from the Broad Institute (http://www.broadinstitute.org/).

### Statistical analysis

We identified potentially differentially expressed genes by comparing read frequencies in the non-normalized, reference isolate cDNA libraries constructed from (i) mycelial cultures, (ii) cultures treated with LPPE, and (iii) spores. To account for unigene redundancy per gene we assessed library-specific read frequencies per UL rather than per unigene. Also, we counted only one read per transcript, and ignored reverse-assembled reads in contigs. After normalizing the read counts, we performed pair wise comparisons of libraries using a modified Fisher's exact test.

## Authors' contributions

UH performed the data analysis and drafted the manuscript. SD designed and performed experiments with the fungi and prepared the mRNA. CIK, ML and HH constructed the cDNA libraries. YW performed the qRT-PCR. TRD and NYL conducted the EST assembly. RAH directed EST sequencing. GR participated in drafting the technical parts of the manuscript. SJMJ, CB and JB conceived and directed the project. All co-authors critically reviewed and edited the manuscript. All authors read and approved the final manuscript.

## Supplementary Material

Additional file 1 **Table S1**. Excel file containing the supplementary table with all unigene locations, their coordinates on the *G. clavigera *genome, the ESTs that mapped to each UL, relevant annotations, and expression analysis results. Click here for file
